# Complications After Thyroidectomy in Children: Lymph Node Dissection Is a Risk Factor for Permanent Hypocalcemia

**DOI:** 10.3389/fendo.2021.717769

**Published:** 2021-09-29

**Authors:** Jesse J. van Rooijen, A. S. Paul van Trotsenburg, Daniël J. van de Berg, Nitash Zwaveling-Soonawala, Els J. M. Nieveen van Dijkum, Anton F. Engelsman, Joep P. M. Derikx, Christiaan F. Mooij

**Affiliations:** ^1^ Department of Pediatric Endocrinology, Emma Children’s Hospital, Amsterdam University Medical Centers, University of Amsterdam, Amsterdam, Netherlands; ^2^ Department of Pediatric Surgery, Emma Children’s Hospital, Amsterdam University Medical Centers, University of Amsterdam and Vrije Universiteit, Amsterdam, Netherlands; ^3^ Department of Surgery, Amsterdam University Medical Centers, University of Amsterdam and Vrije Universiteit, Amsterdam, Netherlands

**Keywords:** thyroid cancer, Graves’ disease, thyroidectomy, hypocalcemia, recurrent laryngeal nerve (RLN) injury, postoperative complications

## Abstract

**Background:**

Thyroidectomy is a treatment option in some benign thyroid disorders and the definitive treatment option for thyroid cancer. As postoperative mortality is extremely rare data on postoperative complications and long-term health consequences are important.

**Objective:**

To evaluate the frequencies of short- and long-term complications, and their risk factors in pediatric patients (0-18 years) who underwent a thyroidectomy in a tertiary children’s hospital.

**Methods:**

A retrospective single center study was performed including all pediatric patients who underwent a thyroidectomy between January 2013 and February 2020.

**Results:**

Forty-eight patients were included in this study (mean age 14.6 years). Twenty-nine total thyroidectomies and 19 hemithyroidectomies were conducted. Thyroid carcinoma was the indication to perform a thyroidectomy in 12 patients, 36 patients underwent a thyroidectomy because of a benign thyroid disorder. Postoperative hypocalcemia was evaluated in patients who underwent a total thyroidectomy. Rapidly resolved hypocalcemia was observed in three patients (10.3%), transient hypocalcemia in 10 patients (34.5%) and permanent hypocalcemia in six patients (20.7%). Permanent hypocalcemia was only seen in patients who underwent a thyroidectomy combined with additional lymph node dissection because of thyroid carcinoma [thyroid carcinoma: OR 43.73, 95% CI (2.11-904.95); lymph node dissection: OR 76.14, 95% CI (3.49-458.98)]. Transient and permanent recurrent laryngeal nerve injury was reported in four (8.3%) and one (2.1%) of all patients, respectively.

**Conclusion:**

Permanent postoperative complications after thyroidectomy are rare in pediatric patients undergoing a thyroidectomy without lymph node dissection. However, in this age group permanent hypocalcemia occurs more frequently after thyroidectomy with additional lymph node dissection because of thyroid cancer. With respect to quality of life, especially of pediatric thyroid cancer patients, reducing this complication is an important goal.

## Introduction

Thyroidectomy is the cornerstone of treatment for pediatric thyroid cancer. Furthermore, it is a definitive treatment option for benign thyroid disorders such as Graves’ disease and benign thyroid nodules (1–6). Prophylactic thyroidectomy can be considered in patients with an increased risk of developing thyroid cancer to prevent the development of malignant disease later in life (7, 8). Mortality after thyroidectomy is very low and the thirty-years survival in pediatric patients with well-differentiated thyroid cancer is 95% (9). Therefore, data on postoperative complications in children are important as these complications may have a significant impact on quality of life (10–12).

Postoperative hypocalcemia and recurrent laryngeal nerve (RLN) injury are complications specific for thyroid surgery. Both can be transient or permanent problems (11, 12). In most cases, postoperative hypocalcemia is caused by hypoparathyroidism due to devascularization or accidental removal of the parathyroid glands (10, 12). Permanent hypocalcemia/hypoparathyroidism requires lifelong treatment with calcium and/or active vitamin D (13, 14). Almquist et al. even reported a twofold increased risk of death in adult patients who developed permanent hypocalcemia due to total thyroidectomy (15). Postoperative RLN injury is caused by damage to the nerve and may result in hoarseness and dysphagia (10, 16). Other general postoperative complications that can be seen after thyroidectomy include hemorrhage, wound infection and keloid formation (12).

To date several studies reported frequencies of complications after pediatric thyroidectomy (1, 10, 11). In this study we aimed to additionally identify possible risk factors for the occurrence of postoperative complications for better counseling of pediatric patients and parents.

## Materials and Methods

### Study Design and Data Collection

A single center retrospective cohort study was conducted in all pediatric patients (age below 18 years) who underwent a thyroidectomy between January 1^st^ 2013 and February 1^st^ 2020. All included patients were treated in the Emma Children’s Hospital, Amsterdam University Medical Centers, a tertiary care hospital for pediatric endocrine disorders including surgical treatment. All pediatric patients who underwent a thyroidectomy were identified in the pediatric endocrinology new patients’ database (started in January 2012). The study was approved by the local Medical Ethical Committee (Amsterdam UMC) (reference number W20_076) and confirmed that the Medical Research Involving Human Subjects act (WMO) does not apply to this study, meaning that formal approval of patients/parents was not needed.

Disease and patient characteristics (age at time of thyroidectomy and gender) were collected for all included patients. The pre- and postoperative diagnosis, preoperative thyroid function parameters and details on the performed surgical procedure (use of intraoperative nerve monitoring, method of vessel ligation technique, number of identified parathyroid glands and parathyroid re-implantation) were collected from the electronic patient charts. For each patient it was evaluated if they were preventively treated with active vitamin D (calcitriol). To evaluate the presence of postoperative complications, data on postoperative calcium levels, signs of RLN injury, hemorrhage or keloid development were collected. Furthermore, the need and reason of postoperatively started calcium and/or active vitamin D treatment was registered. The follow-up duration of each patient was identified.

### Surgical Procedure

Thyroid surgery was always performed by a pediatric surgeon with special interest in endocrine surgery and an endocrine surgeon performing more than 50 thyroidectomies per year. Thyroid surgery in this study was defined as hemithyroidectomy or total thyroidectomy. Total thyroidectomy was subclassified as total thyroidectomy only, total thyroidectomy in two tempi (a hemithyroidectomy followed by a completion thyroidectomy removing the contralateral thyroid lobe), and total thyroidectomy (in two tempi) with central and/or lateral neck dissection. The used method of ligating blood vessels was reported as electrocoagulation (use of energy device) (17) or the use of sutures (tie and knot technique). Intraoperative nerve monitoring was used in most thyroidectomies and data on the reported loss of signal of nerve integrity monitor (NIM; NIM-RESPONSE 3.0, Medtronic, MN, USA) during operation was extracted from the electronic patient chart (18). The vast majority of the patients that underwent a total thyroidectomy were treated with active vitamin D (calcitriol) preoperatively to reduce the risk of postoperative hypocalcemia according to our local protocol. When hypocalcemia did not occur or additional interventions were not needed, active vitamin D usage was stopped at the 16^th^ day after surgery.

### Postoperative Complications

The postoperative complications evaluated within this study were transient and permanent hypocalcemia, transient and permanent RLN injury, postoperative hemorrhage and keloid formation. Some patients developed a postoperative drop in calcium levels which resolved within the first 36 hours after surgery, which we scored as “rapid resolved hypocalcemia”. Superior laryngeal nerve injury was not monitored due to its variable anatomy and difficulty of detecting it (19). The definitions used for the complications are described in detail in **Table 1**.

**Table 1:** Definitions used for the different postoperative complications evaluated in pediatric patients after thyroidectomy.

Postoperative complication	Definition
**Hypocalcemia**	- At least one calcium plasma level < 2.0 mmol/L with clinical signs of hypocalcemia OR - At least one total calcium plasma level < 1.9 mmol/L with or without clinical signs of hypocalcemia AND - Treatment with calcium in addition to perioperative preparation with active vitamin D OR - Post operative start of calcium/active vitamin D without preoperative preparation.
**Rapid resolved hypocalcemia**	- Calcium treatment could be stopped before discharge without prolonged hospitalization OR - Treatment with calcium could be stopped and perioperative active vitamin D scheme could be continued without changes.
**Transient hypocalcemia**	- Required prolonged preoperative supplementation with active vitamin D postoperative to normalize calcium levels OR - Required supplementation with calcium and/or active vitamin D to achieve normal calcium levels up to six months after thyroidectomy.
**Permanent hypocalcemia**	- Required supplementation with calcium and/or active vitamin D longer than six months post thyroidectomy.
**Recurrent laryngeal nerve (RLN) injury**	- Postoperative dysphagia and/or hoarseness AND/OR - Loss of signal of nerve integrity monitor AND - Need of visiting a speech therapist
**Transient RLN injury**	- Symptoms of RLN neuropraxia resolving within six months
**Permanent RLN injury**	- Symptoms of RLN neuropraxia persisting six months after thyroidectomy
**Post-operative hemorrhage**	- Need of re-operation for hemorrhage.
**Keloid**	- Identification of keloid by the surgeon.

Hypocalcemia was only evaluated in those patients who underwent a total thyroidectomy or a completion thyroidectomy due to the predicted very low risk of postoperative hypocalcemia after a hemithyroidectomy. When patients were lost to follow-up within the first six months after thyroidectomy (in most cases because patients were referred to the pediatrician in their local hospital for further follow-up) but were normocalcemic without supplementation or did not have signs of RLN injury during the last visit to our outpatient clinic, they were scored as not having permanent RLN injury or hypocalcemia. None of the patients requiring supplementation because of hypocalcemia were lost to follow up. If patients were lost to follow up within the first six months after thyroidectomy and did not develop signs of keloid at that time, they were excluded for the evaluation of keloid development as keloid can still develop later after surgery (**Table 1**).

### Statistical Analysis

Descriptive statistics were used to describe complication rates and are presented as medians with range. Normality of distribution of data was assessed with the Kolmogorov-Smirnov test. Odds ratios were calculated using a univariate analysis to identify risk factors for postoperative complications. The presence of complications in patients with benign versus malignant disease, and in patients who underwent a total thyroidectomy only versus total thyroidectomy plus lymph node dissection were compared. A p-value of <0.05 was considered as statistically significant. The Haldane-Anscombe correction was used as a zero-cell correction for calculation of odds ratios (20). SPSS Version 25 (SPSS, Chicago, IL, USA) was used to perform statistical analyses.

## Results

### Demographics and Clinical Characteristics

All 48 pediatric patients who underwent a thyroidectomy between January 1^st^ 2013 and February 1^st^ 2020 within the Emma Children’s Hospital, Amsterdam University Medical Centers were included (**Table 2**). The median age at the time of operation was 14.6 years (range: 3.9 – 17.9 years) with a median follow-up duration of 1.6 years (range: 0.1 – 6.0 years). The follow-up duration was 12 to 24 months in 27.1% of the patients (n = 13), and longer than 24 months in 37.5% of the patients (n = 18).

**Table 2:** Demographic, clinical and surgical characteristics of 48 included patients.

Variable	Number (%)
**Demographics**	
Age at time of surgery (median [range], years)	14.6 [3.9 – 17.9]
Female	37 (77.1)
Male	11 (22.9)
**Indications for surgery**	
Graves’ disease	12 (25.0)
Other benign	24 (50.0)
Thyroid carcinoma	12 (25.0)
**Type of surgery**	
Hemithyroidectomy	19 (39.6)
Total thyroidectomy	29 (60.4)
Total thyroidectomy only	18 (37.5)
Total thyroidectomy in two tempi only	2 (4.2)
Total thyroidectomy in two tempi with central neck dissection	1 (2.1)
Total thyroidectomy plus central neck dissection	3 (6.3)
Total thyroidectomy plus central and lateral neck dissection	5 (10.4)
**Pre-operative levels of TSH and free T4**	
TSH (median [range], mU/L)	0.4 [0.0 – 4.4]^a^
Free T4 (median [range], pmol/L)	14.3 [8.1 – 67.9]^a^
**Patients who received perioperative active vitamin D supplementation**	25 (86.2)^b^

a Perioperative TSH and Free T4 levels were measured in 33 patients.

b Since our local guideline only recommends perioperative active vitamin D for a total thyroidectomy, our frequency is based on total thyroidectomies (n = 29).

The majority of the patients were female (n = 37; 77.1%) and underwent a thyroidectomy because of a benign condition (n = 36; 75.0%). The patients with a benign thyroid condition were diagnosed with Graves’ disease (n = 12; 25.0%), benign thyroid nodule(s) (n = 16; 33.3%), Hashimoto’s disease (n = 1; 2.1%), piriform sinus fistula (n = 2; 4.2%), goiter due to Pendred syndrome (n = 1; 2.1%), goiter due to a congenital activating TSH receptor mutation (n = 1; 2.1%) and multiple endocrine neoplasia type 2A without thyroid carcinoma (prophylactic thyroidectomy) (n = 3; 6.3%). Twelve patients (25.0%) underwent a thyroidectomy because of malignancy (papillary thyroid carcinoma in all cases).

Preoperative thyroid function parameters were measured in 33 patients. Thirty-one (93.9%) of them were classified as euthyroid [free T4 levels within the local reference range: 10-23 pmol/L] at the time of surgery. Two patients with Graves’ disease were hyperthyroid at the time of surgery based on their free T4 levels. One of these patients underwent surgery with a preoperative free T4 level of 29.9 pmol/L. The other patient had a free T4 level of 67.9 pmol/L, but was clinically classified as euthyroid because of normalization of T3 levels before surgery due to rapid preoperative preparation with high dose propylthiouracil, propranolol, hydrocortisone and amiodarone (21).

### Surgical Details

Forty-eight patients were operated 51 times (**Table 2**). The thyroidectomies were classified as hemithyroidectomy (n = 19; 39.6%) or total thyroidectomy (n = 29;60.4%). Two of the total thyroidectomies were performed in two tempi because of the histopathological diagnosis thyroid cancer. In nine of the twelve patients diagnosed with thyroid carcinoma additional lymph node dissection was performed according to the current guidelines (22). In one patient with thyroid cancer a hemithyroidectomy was performed; due to comorbidity and the small size of the T1 tumor follow-up by ultrasonography was preferred over a completion thyroidectomy. Postoperative mortality was not seen in the studied cohort.

In the vast majority of thyroidectomies vessel ligation was performed by electrocoagulation (LigaSure, n = 41; 85.4%). In five thyroidectomies (10.4%) vessel ligation was performed using the tie-and-knot technique. In two (4.2%) thyroidectomies the used vessel ligation method was not specified within the surgery record.

NIM was used in 47 out of 48 thyroidectomies (97.9%) to monitor RLN integrity. In most thyroidectomies (75.0%) no loss of signal was perceived by the end of surgery. In seven thyroidectomies (14.6%) a unilateral loss of signal occurred. In four (8.3%) of the thyroidectomies the quality of NIM signal was not described in the surgical record.

Pathology reports of all patients who underwent a total or completion thyroidectomy were evaluated. In 17 (58.6%) of all 29 pathology reports the number of found parathyroid glands was clarified. In four (13.8%) pathology reports it was reported that parathyroid tissue was not present in the specimen. In seven (24.1%) specimens one parathyroid gland was found, in five (17.2%) specimens two parathyroid glands were found, and in one specimen four parathyroid glands were found (3.4%). In the remaining 12 (41.4%) pathology reports the number of identified parathyroid glands was not specified, probably because in those cases no parathyroid glands were removed. Auto-transplantation of parathyroid glands was carried out in five of 29 total thyroidectomies, in two of these cases parathyroid tissue was not present in the pathology specimen.

### Postoperative Complications

The frequencies of postoperative complications are reported in **Tables 3A**, **B**. Preoperative calcium levels were normal in all patients who underwent a total or completion thyroidectomy. Postoperative calcium levels were only evaluated in the patients who underwent a total or completion thyroidectomy (n=29; **Table 3A**). Postoperative hypocalcemia (rapid resolved, transient or permanent) occurred in a total of 19 patients (65.5%). Rapid resolved hypocalcemia was reported in three patients (10.3%), who did not need treatment, and were all discharged from the hospital three days after surgery with normal calcium levels. Transient hypocalcemia was reported in ten patients (34.5%) of which three patients underwent auto-transplantation of a parathyroid gland during thyroidectomy. Permanent hypocalcemia was present in six patients (20.7%), and all these six patients had undergone lymph node dissection because of metastatic thyroid cancer (54.5% of all patients with thyroid cancer; 66.7% of all patients who underwent additional lymph node dissection). One of these six patients underwent an auto-transplantation of one parathyroid gland. Due to the low number of patients who did not receive preoperative active vitamin D, we did not evaluate the effect of perioperative active vitamin D treatment on the frequency of postoperative hypocalcemia. Transient hypocalcemia was seen in patients who underwent a total thyroidectomy because of papillary thyroid carcinoma (n = 3; all three patients underwent a lymph node dissection), Graves’ disease (n = 4), goiter due to a congenital activating TSH receptor mutation (n = 1), goiter due to Pendred syndrome (n = 1) and a prophylactic thyroidectomy (n = 1).

**Table 3A:** Postoperative hypocalcemia in pediatric patients who underwent a total thyroidectomy.

	Rapid resolved hypocalcemia (n)	Transient hypocalcemia (n)	Permanent hypocalcemia (n)
All patients (n = 29)^a^	3	10	6
**Total thyroidectomy only (n = 20)**	3	7	0
­ Graves’ disease (n = 12)	3	4	0
­ Other benign (n = 6)	0	3	0
­ Thyroid carcinoma (n = 2)	0	0	0
**Total thyroidectomy plus lymph node dissection in metastatic thyroid carcinoma (n = 9)**	0	3	6

a This is a subgroup of all patients included in the study (n = 48) in whom postoperative calcium levels were measured.

**Table 3B:** Postoperative complications in pediatric patients who underwent a thyroidectomy.

	Transient RLN (n)	Permanent RLN (n)	Postoperative hemorrhage (n)	Keloid ^b^ (n)
**All patients (n = 48)**	4	1	0	8
**Total thyroidectomy only (n = 20)**	1	0	0	3
­ Graves’ disease (n = 12)	1	0	0	2 ^c^
­ Other benign (n = 6)	0	0	0	1
­ Thyroid carcinoma (n = 2)	0	0	0	0
**Total thyroidectomy plus lymph node dissection in metastatic thyroid carcinoma (n = 9)**	1	0	0	2
**Hemithyroidectomy because of benign thyroid disorder (n = 18)**	2	1	0	3 ^d^
**Hemithyroidectomy because of thyroid carcinoma ^a^ (n = 1)**	0	0	0	0

^a.^In one patient with thyroid carcinoma a hemithyroidectomy was performed and therefore no postoperative calcium levels were measured.

^b.^Six patients had to be excluded due to follow-up period < six months.

^c.^Four patients had to be excluded due to follow-up period < six months.

^d.^Two patients had to be excluded due to follow-up period < six month.

RLN injury was observed in five of 48 patients (10.4%). In the majority of these cases the RLN injury was transient (80%; n=4). Transient RLN injury was seen in patients who underwent a thyroidectomy because of Graves’ disease (n=1), a benign thyroid nodule (n=2) or papillary thyroid cancer (n=1). In three of these four patients, symptoms disappeared within three months after surgery, in one patient symptoms resolved five months post-surgery. Permanent RLN injury was seen in only one patient who underwent a hemithyroidectomy because of a benign thyroid nodule (2.1%), in this patient a loss of NIM signal was reported during surgery. NIM signal was adequate during thyroidectomy in all patients without signs of RLN injury. Keloid was present in eight patients (19.0%), none of these patients underwent a thyroidectomy in two tempi. No cases of postoperative hemorrhage were seen. One patient had to undergo a re-operation due to an abscess.

### Risk Factors for Postoperative Complication

Permanent hypocalcemia only occurred in patients who underwent a thyroidectomy because of a malignancy (**Table 3A**). When comparing patients who underwent a thyroidectomy with patients who underwent a thyroidectomy plus lymph node dissection, the lymph node dissection was associated with permanent postoperative hypocalcemia [OR 76.14, 95%CI (3.49-458.98), (p<0.05)]. Patients who underwent a thyroidectomy because of thyroid cancer seemed to have a higher risk of permanent postoperative hypocalcemia compared to patients with a benign thyroid disorder [OR 43.73, 95%CI (2.11-904.95), (p<0.05)], although this increased risk seems to be attributed to the performance of the additional lymph node dissection in patients with metastatic thyroid cancer.

No other risk factors for transient or permanent complications were identified as all other calculated ORs were not statistically significant (data not shown). However, rapid resolved hypocalcemia tended to be more frequent in patients with Graves’ disease (three out of twelve patients) versus other benign thyroid disorders (zero out of six patients).

## Discussion

Mortality after pediatric thyroidectomy is very rare, but complications of thyroidectomy do occur [6,9,10,11]. In this study the frequencies of short- and long-term complications in pediatric patients (0-18 year) who underwent a thyroidectomy were evaluated and risk factors associated with postoperative complications were identified. Transient postoperative complications were observed relatively frequent in this study. Permanent RLN injury was extremely rare and only seen in one patient (2.1%). Permanent hypocalcemia was not seen in pediatric patients who underwent a thyroidectomy because of a benign thyroid condition, neither in patients who underwent a thyroidectomy *without* additional lymph node dissection because of thyroid cancer. Overall, permanent hypocalcemia was regularly seen after total thyroidectomy because of thyroid cancer and only in those patients who underwent a central or lateral neck dissection because of metastatic disease. The performance of an additional central or lateral neck dissection (because of thyroid cancer) was associated with an increased risk of postoperative permanent hypocalcemia.

In 2015, we reported quality of life and clinical outcomes after pediatric thyroidectomy carried out between 2000 and 2012 (n=40) (10). In that study period a 10% frequency of transient RLN paralysis and a 32.5% frequency of transient hypocalcemia was reported. No cases of permanent RLN injury were reported, while 12.5% of the studied patients suffered from permanent hypocalcemia (both patients with thyroid cancer or a benign thyroid disorder). These frequencies are in line with those found in this study showing a consistent outcome of thyroidectomy in our center over the time.

Postoperative hypocalcemia was the most frequently reported postoperative complication in our cohort of pediatric patients after a total thyroidectomy. The reported frequency of transient hypocalcemia in our cohort (34.5%) is in the range of previously reported frequencies of transient hypocalcemia in pediatric patients after thyroidectomy (5.7% – 49.3%) (1, 10, 11, 23–25). The relatively high rate of transient hypocalcemia and thus temporary calcium supplementation in this study may be explained by individual institutional protocols and policies. This may have resulted in an unnecessary start of treatment with calcium/active vitamin D. Furthermore, in our experience some patients experience a very short drop in postoperative calcium levels without causing prolonged hospitalization or longer duration of treatment with calcium or active vitamin D. Therefore, we classified these patients as having ‘rapid resolved hypocalcemia’. The occurrence of rapid resolved hypocalcemia was 10.3% (n = 3 out of 29 patients) and is of small clinical relevance. Uniformity in diagnosing and treating postoperative hypocalcemia is needed.

Permanent hypocalcemia occurred in 20.7% of patients in this study which fits within the previously reported frequencies in other studies (0.6% - 21.7%) (1, 10, 11, 23–25). In adults the incidence is lower compared to children (1.0% -12.5%) (26–28). In our cohort permanent hypocalcemia only occurred in patients who underwent a total thyroidectomy plus lymph node dissection because of thyroid cancer. The results suggest that the additional lymph node dissection, if indicated, in pediatric thyroid cancer patients may be the main cause of permanent hypoparathyroidism as no cases of permanent hypocalcemia are seen after total thyroidectomy without additional lymph node dissection. It has been shown previously in adults that central neck lymph node dissection is an independent risk factor for permanent hypoparathyroidism (29–31). The increased risk of postoperative permanent hypocalcemia in patients who underwent additional lymph node dissection is possibly caused by devascularization or unintentional resection of parathyroid glands (24). However, data on identification and/or re-implementation of parathyroid glands can give more information on the risk of development of postoperative hypocalcemia. De Jong et al. found a higher incidence of postoperative hypocalcemia when a lower number of parathyroid glands remained *in situ* (11). Due to the retrospective character of this study and the fact that data on the number of identified parathyroid glands during surgery were not fully reported in the medical charts, we could not prove a relation between the number of parathyroid glands that remained *in situ* and permanent hypocalcemia.

Several factors may reduce the risk of postoperative hypoparathyroidism in the future. First of all, pediatric thyroidectomies should be performed by high-volume dedicated pediatric endocrine surgeons (6), as is the case in our children’s hospital. Furthermore, meticulous capsular dissection and the intra-operative localization of the parathyroid glands by fluorescence may reduce the risk of the accidental removal or devascularization of the parathyroid glands (32, 33). Intra-operative localization of the parathyroid glands is of great importance during a central neck dissection to prevent damaging the parathyroid glands. As permanent hypocalcemia requires lifelong treatment with active vitamin D and/or calcium the prevention of this complication would improve the long-term health outcome of especially pediatric patients undergoing a total thyroidectomy.

RLN injury is the second most frequent reported complication after thyroidectomy. The occurrence of transient (8.3%) and permanent (2.1%) RLN injury in our study was in line with other studies (0.6%- 10% and 0% - 1.6% respectively) (1, 10, 23, 24). The golden standard to detect RLN injury is the use of laryngoscopy revealing vocal cord paralysis (18). Due to the invasive character of laryngoscopy, it is not routinely used in pediatric patient care after thyroidectomy (1, 10, 23, 24). As we believe transient (and permanent) RLN injury is only clinically relevant if symptoms like hoarseness persist after the direct postoperative period, we only classified patients with RLN injury when they were referred to a speech therapist (34). A key aspect in avoiding RLN injury during the operation is identification of the RLN (35). In the majority of thyroidectomies in this study NIM was used, which helps to identify the nerve more easily and guards nerve function to prevent injury (36). Future improvement of techniques identifying the RLN may even further reduce the risk of RLN injury after thyroidectomies in children.

The reported frequency of keloid in our cohort (19%) was higher compared to previous studies (0% - 7%) (37, 38). As this frequency is relatively high, we believe it is important to counsel patients about the risk of keloid development as it may cause pain, itchiness and cosmetic disturbance (39). None of the patients in our cohort developed a postoperative hemorrhage. In most of our patients LigaSure was used as technique for vessel ligation. Since previous studies showed better hemostasis with LigaSure compared to conventional sutures this may explain the absence of postoperative hemorrhage in our cohort (40).

This study has several limitations. Due to the retrospective nature of the study completeness of data differed per patient. In addition, treatment strategies for hypocalcemia were not uniform. In order to specify the occurrence and duration of postoperative hypocalcemia, we used specific criteria (**Table 1**). The used definitions for most of the complications reported in this study are subjective. This may have influenced the reported frequencies in this study. It has been shown that parathyroid function may recover more than six months after total thyroidectomy (41), as a result the reported frequency of permanent hypocalcemia in our study may have been overestimated. Another important limitation of this study is that postoperative parathyroid hormone (PTH) levels were not measured postoperatively as part of standard care. As data on postoperative PTH levels were lacking we were unable to prove that postoperative hypocalcemia is caused by postoperative hypoparathyroidism. However, the fact that calcium levels were normal preoperatively in all patients indicates that there were no signs of preexisting hypocalcemia. To identify the cause of postoperative hypocalcemia after a total thyroidectomy we suggest incorporating postoperative PTH measurements into the standard biochemical follow-up after a total thyroidectomy. Furthermore, the number of patients included in this study is relatively low causing a lack of power for subgroup analysis, a high frequency of complications in certain subgroups and high calculated ORs with wide confidence intervals. The fact that thyroidectomy in children is a rare procedure has led to this relatively low number of included patients. However, the reported cohort from our tertiary referral center is a relatively large cohort compared to previously published studies.

In conclusion, this study showed that permanent complications after thyroidectomy without additional lymph node dissection are rare. However, permanent hypocalcemia is relatively common in pediatric patients with thyroid cancer who require an additional lymph node dissection. Thyroid cancer and especially the performance of a lymph node dissection were associated with an increased risk of permanent hypocalcemia after pediatric thyroidectomy, as already has been shown in adults (27). Further improvement of identification techniques of both the parathyroid glands and RLN are important to reduce risk of postoperative complications and potentially reduce the impact of life long postoperative morbidities on the quality of life, especially in patients with pediatric thyroid cancer.

## Data Availability Statement

The original contributions presented in the study are included in the article. Further inquiries can be directed to the corresponding author.

## Ethics Statement

The studies involving human participants were reviewed and approved by Medical Ethical Committee (Amsterdam UMC). Written informed consent from the participants’ legal guardian/next of kin was not required to participate in this study in accordance with the national legislation and the institutional requirements.

## Author Contributions

JvR performed the data collection and analysis under supervision of JD and CM. JvR, JD, and CM took the lead in writing the manuscript. AvT, NZ-S, ENvD, AE, JD, and CM were all involved in the clinical care for the patients included in this study. AvT, DvdB, NZ-S, ENvD, and AE critically reviewed and improved the manuscript. All authors contributed to the article and approved the submitted version.

## Funding

This research was financially supported by a *Steun Stichting Emma Kinderziekenhuis* grant (project number WAR2020-10).

## Conflict of Interest

The authors declare that the research was conducted in the absence of any commercial or financial relationships that could be construed as a potential conflict of interest.

## Publisher’s Note

All claims expressed in this article are solely those of the authors and do not necessarily represent those of their affiliated organizations, or those of the publisher, the editors and the reviewers. Any product that may be evaluated in this article, or claim that may be made by its manufacturer, is not guaranteed or endorsed by the publisher.
